# Development of a predictive nomogram for switching immunosuppressive drugs in pediatric liver transplant recipients

**DOI:** 10.3389/fped.2023.1226816

**Published:** 2023-10-19

**Authors:** Guangxiang Gu, Tao Zhou, Zhipeng Zong, Jianjun Zhang

**Affiliations:** ^1^ Department of Liver Surgery, Renji Hospital, School of Medicine, Shanghai Jiao Tong University, Shanghai, China; ^2^ Department of Liver Transplantation, Sun Yet-sen Memorial Hospital, Sun Yat-sen University, Guangzhou, China

**Keywords:** immunosuppressive drugs, pediatric liver transplantation, nomogram, tacrolimus, cyclosporin

## Abstract

**Background:**

Tacrolimus (TAC) is the preferred calcineurin inhibitor (CNI) for pediatric liver transplant recipients. However, some recipients may not achieve the desired therapeutic window concentration of TAC, leading to poor prognosis. This study aimed to develop a clinical model that can predict the effectiveness of TAC in pediatric liver transplant recipients and help clinicians quickly identify cyclosporin as an alternative.

**Methods:**

We retrospectively analyzed data from 2,032 pediatric liver transplant recipients who underwent surgery at Renji Hospital, Shanghai Jiaotong University School of Medicine between 2006 and 2019. Demographic, comorbidity and pre-operative laboratory data were collected, and a nomogram was constructed using multivariate logistic regression analysis to estimate the risk of poor therapeutic outcomes for TAC-based immunosuppression.

**Results:**

The constructed nomogram included seven parameters, namely recipient CYP3A4 genotype, pre-transplant cholangitis, GRWR, spleen long diameter, serum albumin, graft volume reduction, and donor CYP genotype. The nomogram showed good discriminative ability with an area under the receiver operating characteristic curve (AUC) of 74.5% and good calibration. Decision curve analysis indicated a high potential clinical application of the model.

**Conclusion:**

This simple clinical model effectively predicts the risk of poor therapeutic outcomes in pediatric liver transplant recipients who receive TAC-based immunosuppression. Clinicians can use the model to identify cyclosporin as an alternative quickly, potentially improving patient prognosis.

## Introduction

The routine and widespread utilization of immunosuppressive (IS) agents has contributed significantly to the consistent enhancements in post-transplant survival rates (1). However, in the context of pediatric liver transplantation (LT), achieving the desired therapeutic concentration window of tacrolimus (TAC) poses a distinctive challenge, carrying substantial implications for patient outcomes. The deleterious consequences stemming from suboptimal TAC levels are manifest in the diminished occurrences of severe acute rejection and graft loss attributable to rejection, with more than 63% of late mortality post pediatric liver transplantation (LT) attributed to non-hepatic factors (2).

Calcineurin inhibitors (CNIs), such as tacrolimus (TAC) or cyclosporine (CsA), corticosteroids, and antimetabolites (most commonly mycophenolic acid), are currently the most commonly used drugs for LT immunosuppression (3). Among these, CNIs, particularly TAC, are the primary choice, with over 80% of pediatric LT recipients undergoing TAC-based immunosuppression (4). However, the long-term use of CNIs may lead to substantial adverse effects, including malignancy, infection, metabolic disorders, and organ toxicities (5, 6). Consequently, personalized therapeutic strategies should be employed to mitigate these adverse effects.

TAC is the preferred CNI drug after pediatric LT, due to its higher potency and superior post-transplant survival rates than CsA (4). Although the side effects of TAC and CsA are similar, including hypertension, nephrotoxicity, neurotoxicity, and lipid metabolic disorders, they have different immunological mechanisms and pharmacokinetics (7). Recipients may develop different benefit and harm profiles with TAC or CsA treatment. Clinicians typically consider switching from TAC to CsA when recipients develop severe side effects or experience unsatisfactory efficacy during TAC therapy (Table 1). In certain recipient populations, CsA may present a more advantageous alternative.

**Table 1:** Comparison between CsA and TAC.

CNIs	Superiority compared to TAC/CsA	Conditions may consider switching TAC to CsA
CsA	Lower rate of new-onset diabetes after transplant (NODAT) (9) May have lower neurotoxicity (10) Reduced the risks after liver transplantation of death, graft loss, acute rejection and steroid-resistant rejection (9, 11)	The serum concentration of TAC is low and unable to reach the target serum concentration after increasing the dose of TAC. Severe complications developed such as posterior reversible encephalopathy syndrome (PRES), seizures and some other CNS symptoms (12, 13).
TAC	Easier to achieve the balance between efficacy and side effects (9)	

CNIs, calcineurin inhibitors; CsA, cyclosporine; TAC, tacrolimus; CNS, central nervous system.

Therefore, it is essential to choose IS drugs based on pre-transplant and/or intraoperative risk factors (8). In this retrospective study of a large sample of pediatric LT recipients, we aimed to identify risk factors for switching IS drugs and construct a simple clinical model using common clinical features to predict the risk of unfavorable treatment outcomes for recipients undergoing a TAC-based IS regimen and enable clinicians to make informed decisions and improving patient care.

## Methods

### Patients and study design

This study included patients who underwent pediatric liver transplantation (LT) at Renji Hospital, Shanghai Jiaotong University School of Medicine from 2006 to 2019. Patients who were lost to follow-up or died within the first month after the operation were excluded. No organs from executed prisoners were transplanted and reported in this study. Ethical approval was granted by the institutional human research committee (Ethics Committee of Renji Hospital) and the study adhered to the ethical guidelines of the 1975 Declaration of Helsinki. Written informed consent was obtained from the parents of each patient included in the study. Follow-up continued until the study's termination date in December 2019.

To dissect the determinants influencing the switching of immunosuppression (IS) drugs after pediatric LT, the patient cohort was divided into two groups: those who switched IS drugs and those who did not. Demographic characteristics, comorbidities, preoperative laboratory parameters, and post-transplantation outcomes were compared between the two groups.

### Variables collected

Clinicopathological variables included recipient and donor age and body weight at LT, gender, cytochrome P450 3A4(CYP3A4) genotype, recipient growth status, spleen length, primary disease of the recipient, pre-transplantation complications, recipient Child-Pugh score, pediatric end-stage liver disease (PELD) score, graft recipient body ratio (GRWR), and surgical type. Preoperative laboratory assessments included serum albumin, bilirubin, international normalized ratio (INR), and prothrombin time (PT). Post-transplantation events included acute rejection within 3 months after pediatric LT, IS drug protocols, complications, and mortality.

### Follow-up

Patients were followed up weekly for the first 3 months after discharge, then every 2 weeks from the fourth to the sixth month, and monthly after 6 months. Routine tests included liver function, viral infection, and blood concentration of immunosuppressants (TAC or CsA). Liver ultrasound was performed at least once every 3 months.

### Immunosuppression protocol after pediatric LT

The primary immunosuppressive agents were TAC, CsA, corticosteroids, and mycophenolate mofetil (MMF). Steroids were administered intravenously and gradually tapered to oral glucocorticoids during the first week after LT. The initial TAC dose was 0.1–0.15 mg/kg/day with a target blood concentration of 8–12 ng/ml in the first month, 7–10 ng/ml between the 2nd and 6th month, 5–8 ng/ml between the 7th and 12th month, and maintained at 5 ng/ml according to liver function after 1 year. The TAC dosage was adjusted based on liver function and blood concentration. MMF was added or TAC was switched to CsA if the blood concentration of TAC was low and the target concentration could not be reached after increasing the TAC dose or severe side effects occurred.

### Statistical analysis

Continuous variables with normal distribution were expressed as mean ± standard deviation, and non-continuous variables were expressed as median and interquartile range (IQR). Categorical variables were analyzed using a chi-square test and expressed as numbers and proportions (%). Continuous variables were analyzed using a *t*-test or Wilcoxon signed-rank test. Statistical analysis was performed using SPSS (IBM, version 26) and R (R Foundation for Statistical Computing).

Variables that were found to be statistically significant (*p* < 0.05) using univariate logistic regression analysis were selected as candidate predictors for the prediction model. After an AIC-stepwise selection process, risk factors were identified using multivariate logistic analysis. Finally, a nomogram was constructed using these risk factors to predict the risk of poor treatment outcomes for recipients who receive an IS protocol based on TAC and may switch to CsA. The established nomogram was evaluated using calibration curves, and its discriminative performance was evaluated using the area under the receiver operating characteristic (ROC) curve (AUC). For internal validation, bootstrap was conducted, and calibration plots were created to compare the predicted survival probabilities and actual probabilities. The clinical usefulness of the nomogram was assessed using decision curve analysis (DCA).

## Results

### Patient characteristics and outcomes

In this study, a total of 2,032 recipients were included, with 1,687 recipients receiving a protocol of IS after pediatric LT and not switching IS drugs. On the other hand, 345 recipients switched IS drugs under the guidance of clinicians midway after pediatric LT. Demographics and clinical characteristics of patients with switching and non-switching IS drugs were listed in Table 2, and significant differences were found in various factors including recipient CYP3A4 genotype (*p* < 0.001), cholangitis before LT (*p* = 0.029), serum albumin (*p* = 0.044), surgical type (*p* = 0.018), graft volume reduction during the operation (*p* = 0.008), types of IS drugs (*p* < 0.001), the addition of MMF (*p* < 0.001), donor CYP3A4 genotype (*p* < 0.001), and donor age at LT (*p* = 0.021) between the two groups (Table 2).

**Table 2:** Characteristics of the switching IS drugs patients and no switching IS drugs patients.

Factor	No switching IS drugs group	Switching IS drugs group	*p* value
Number	1,687	345	
Recipient age at LT, median (IQR), months	8.00 [6.00, 17.00]	9.00 [6.00, 17.00]	0.163
Recipient gender, female	917 (54.4)	174 (50.4)	0.203
Recipient weight at LT, median (IQR), kg	7.70 [6.50, 10.00]	7.80 [6.90, 10.00]	0.206
Growth retardation	1,067 (63.2)	225 (65.2)	0.528
Recipient CYP genotype			<0.001
AA	108 (6.4)	71 (20.6)	
AG	555 (32.9)	181 (52.5)	
GG	1,024 (60.7)	93 (27.0)	
Primary disease at transplantation			0.325
Acute liver failure	8 (0.5)	5 (1.4)	
Cholestatic liver disease	1,502 (89.0)	299 (86.7)	
Metabolic liver disease	124 (7.4)	30 (8.7)	
Neoplastic disease	24 (1.4)	4 (1.2)	
Re-transplantation	20 (1.2)	4 (1.2)	
Vascular disease	9 (0.5)	3 (0.9)	
Complications before transplantation			
History of heart disease	221 (13.1)	43 (12.5)	0.816
Portal hypertension	732 (43.4)	136 (39.4)	0.194
Gastrointestinal bleeding	258 (15.3)	57 (16.5)	0.622
Cholangitis	590 (35.0)	99 (28.7)	0.029
Ascites	1,057 (62.7)	225 (65.2)	0.402
GRWR, median (IQR)	3.14 [2.50, 3.83]	3.28 [2.57, 3.93]	0.065
Spleen long diameter (IQR), millimeter	90.00 [76.00, 107.00]	93.00 [80.00, 105.00]	0.144
INR, median (IQR)	1.30 [1.10, 1.67]	1.29 [1.12, 1.62]	0.911
Albumin, median (IQR), g/dl	3.49 [3.10, 3.92]	3.43 [3.05, 3.84]	0.044
TB, median (IQR)	12.90 [3.50, 20.30]	12.60 [3.30, 19.20]	0.663
PT, median (IQR)	14.70 [12.50, 19.00]	14.60 [12.70, 18.50]	0.901
Child-Pugh score at transplantation, median (IQR)	9.00 [7.00, 10.00]	9.00 [7.00, 10.00]	0.575
PELD score, median (IQR)	18.00 [11.00, 27.00]	19.00 [12.00, 26.00]	0.675
Surgical type			0.018
SLT	86 (5.1)	14 (4.1)	
OLT	213 (12.6)	63 (18.3)	
LDLT	1,388 (82.3)	268 (77.7)	
Graft volume reduction	81 (4.8)	5 (1.4)	0.008
IS drugs			<0.001
TAC	1,679 (99.5)	71 (20.6)	
CsA	8 (0.5)	274 (79.4)	
Addition of MMF	994 (58.9)	293 (84.9)	<0.001
Donor CYP genotype			<0.001
AA	217 (12.9)	83 (24.1)	
AG	699 (41.4)	198 (57.4)	
GG	771 (45.7)	64 (18.6)	
Donor sex (female)	917 (54.4)	174 (50.4)	0.203
Donor age at LT, median (IQR), years	29.00 [25.00, 34.00]	28.00 [23.00, 33.00]	0.021
Donor BMI, median (IQR)	21.90 [19.40, 24.20]	21.20 [18.90, 24.10]	0.06

Data are presented as *n* (%) unless otherwise indicated. BMI, body mass index; IQR, interquartile range; INR, international normalized ratio; TB, total bilirubin; PT, prothrombin time; PELD, pediatric end-stage liver disease; LT, liver transplantation; SLT, split liver transplantation; OLT, orthotopic liver transplantation; LDLT, living donor liver transplantation; IS, immunosuppression; MMF, mycophenolate mofetil.

Patients in the switching IS drugs group had a higher rate of acute rejection within 3 months after LT and a higher rate of developing mental, neurological, and urinary complications after LT. However, they had a lower mortality rate and a lower rate of developing portal vein complications and post-transplant lymphoproliferative disorder (PTLD) than patients in the non-switching IS drugs group (Table 3).

**Table 3:** Outcomes of switching IS drugs patients and no switching IS drugs patients.

	No switching IS drugs group	Switching IS drugs group	*p* value
Number	1,687	345	
Death	139 (8.2)	11 (3.2)	0.005
Viral infection status (positive)			
EBV	911 (54.0)	182 (52.8)	0.716
CMV	463 (27.4)	112 (32.5)	0.069
HBV	91 (5.4)	13 (3.8)	0.265
Acute rejection in 3 months after LT	385 (22.8)	171 (49.6)	<0.001
Vascular complications			
Portal vein	88 (5.2)	8 (2.3)	0.03
Hepatic artery	31 (1.8)	3 (0.9)	0.295
Respiratory complications	455 (27.0)	87 (25.2)	0.546
Digestive complications	366 (21.7)	58 (16.8)	0.257
Urinary complications	98 (5.8)	31 (9.0)	0.037
Mental and neurological complications	33 (2.0)	16 (4.6)	0.006
Hematological complications	189 (11.2)	47 (13.6)	0.236
Skeletal complications	38 (2.3)	7 (2.0)	0.955
Allergy or urticaria	398 (23.6)	65 (18.8)	0.065
PTLD	84 (5.0)	8 (2.3)	0.043

EBV, Epstein–Barr virus; CMV, cytomegalovirus; HBV, hepatitis B virus; PTLD, post-transplant lymphoproliferative disorder.

### Selection of predicting factors associated with switching IS drugs

Univariate logistic analysis revealed multiple factors significantly associated with the risks of switching IS drugs, including recipient CYP3A4 genotype, cholangitis before LT, GRWR, INR, serum albumin, graft volume reduction, the addition of MMF, donor age at LT, donor CYP3A4 genotype, acute rejection within 3 months after LT, portal vein complications, urinary complications, mental and neurological complications, and PTLD (Table 4). In multivariate logistic analysis, seven potential predictors were identified, including recipient CYP3A4 genotype, cholangitis before LT, GRWR, spleen long diameter, serum albumin, graft volume reduction, and donor CYP3A4 genotype.

**Table 4:** Risk factors associated with switching IS drugs.

	Univariate analysis			Multivariate analysis		
	HR	CI	*p*-value	HR	CI	*p*-value
Recipient sex	1.1366	0.9015–1.4332	0.2787			
Recipient age at LT	0.9997	0.9959–1.0033	0.8886			
Recipient CYP genotype						
AA	REF			5.4196	3.6850–7.9743	<0.001
AG	0.4961	0.3524–0.7008	<0.001	3.0827	2.3337–4.0939	<0.001
GG	0.1381	0.0957–0.1995	<0.001	REF		
Complications before transplantation						
History of heart disease	0.9445	0.6586–1.3268	0.7487			
Portal hypertension	0.849	0.6692–1.0741	0.1746			
Gastrointestinal bleeding	1.0962	0.7951–1.4901	0.5658			
Cholangitis	0.7483	0.5785–0.9616	0.0251	1.3556	1.0371–1.7825	0.0275
Ascites	1.1175	0.8785–1.4274	0.3691			
GRWR	1.1222	1.0067–1.2495	0.0363	1.1558	1.0305–1.2954	0.013
Spleen long diameter (IQR), millimeter	1.0006	0.9965–1.0046	0.0756	1.393	1.0734–1.8141	0.0132
INR, median (IQR)	0.9892	0.9504–1.0241	0.0442			
Albumin, median (IQR)	0.8294	0.6901–0.9935	0.0442	0.7915	0.6551–0.9524	0.0143
TB, median (IQR)	0.9982	0.9873–1.0089	0.7451			
PT, median (IQR)	0.9993	0.9949–1.0031	0.7315			
Child-Pugh score at transplantation, median (IQR)	1.0157	0.9618–1.0729	0.5757			
PELD score, median (IQR)	0.9996	0.9912–1.0078	0.9257			
Surgical type						
SLT	REF					
OLT	1.8169	0.9916–3.5323	0.0636			
LDLT	1.1861	0.686–2.2065	0.564			
Graft volume reduction	0.2916	0.102–0.6552	0.008	3.6641	1.5888–10.6458	0.0063
Addition of MMF	3.9284	2.9046–5.411	<0.001			
Donor sex	1.1704	0.9281–1.4758	0.1834			
Donor age at LT	0.9876	0.9781–0.9975	0.0131			
Donor BMI	0.9764	0.9495–1.0041	0.0943			
Donor CYP genotype						
AA	REF			2.8655	1.9599–4.2005	<0.001
AG	0.7406	0.551–1.0009	0.0483	2.3615	1.732–3.252	<0.001
GG	0.217	0.1511–0.3103	<0.001	REF		

NA, not available; REF, reference.

### Construction of prediction nomogram

**Figure 1:**
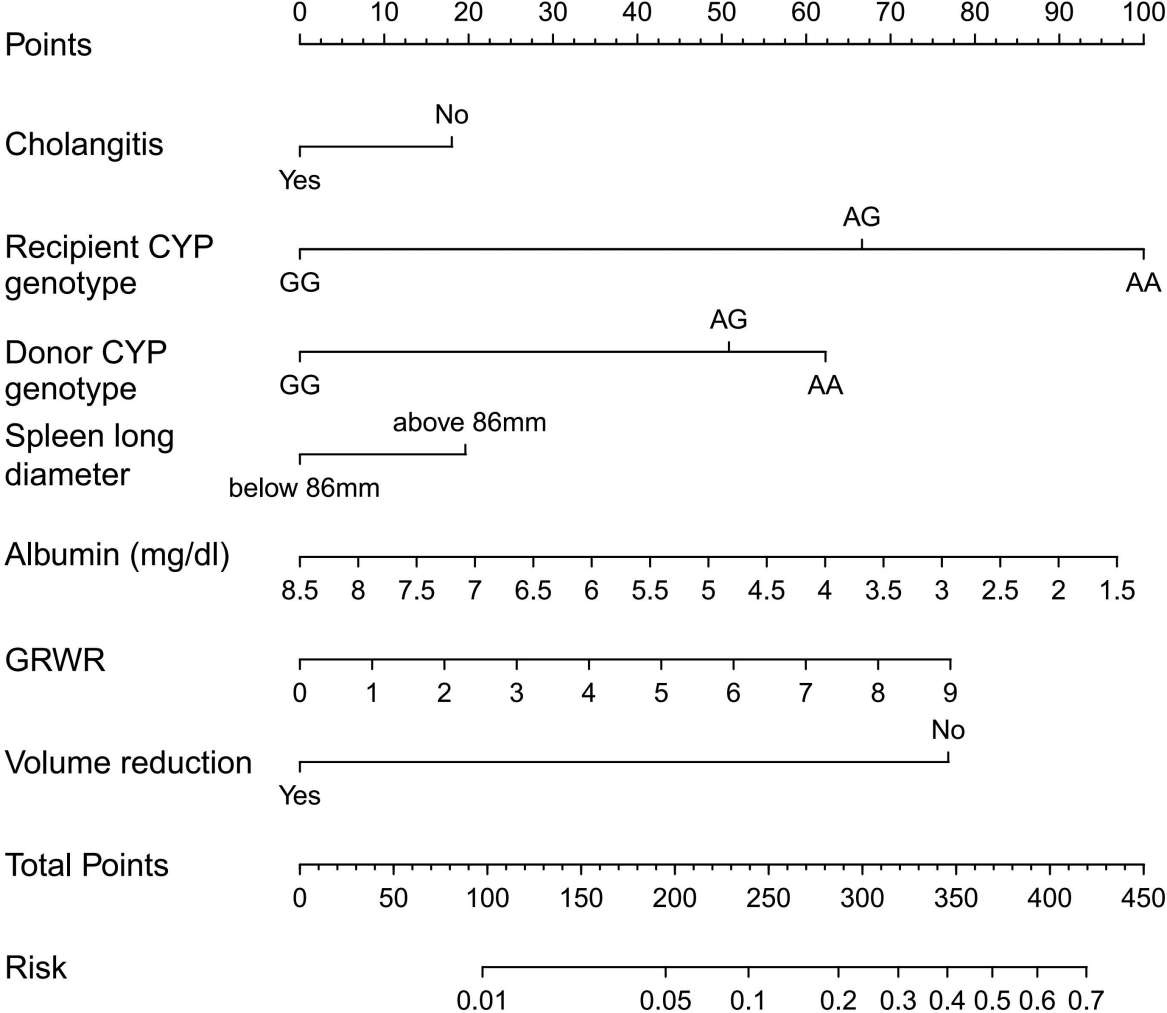
Nomogram for predicting the risk of poor curative effects in recipients receiving an IS protocol based on TAC. A verticle line could be drawn from the value to the top points scale to determine the number of points that assigned by that variable value. Then, the points from each variable value were summed. The sum on the total points scale was located and vertically projected onto the bottom axis, and then the probability of switching IS drugs were obtained.

Based on the multivariate logistic analysis results listed in the previous section and combined with an AIC stepwise model selection and clinical consideration, a total of 7 variables were selected to construct a visualized nomogram model (Figure 1). The length of the variable axis visually represents the relative contribution of each predictor, with recipient CYP3A4 genotype of AA and low serum albumin making the most substantial contribution to the model. The nomogram assigns the probability of switching IS drugs by summing the scores of each risk factor on the points scale. The bottom scale shows the risk of poor curative effects for those recipients receiving an IS protocol based on TAC by the total score. Higher scores indicate a poorer prognosis, promting clinicians to consider initiating CsA instead of TAC within the protocol.

### Validation of nomogram performance

**Figure 2:**
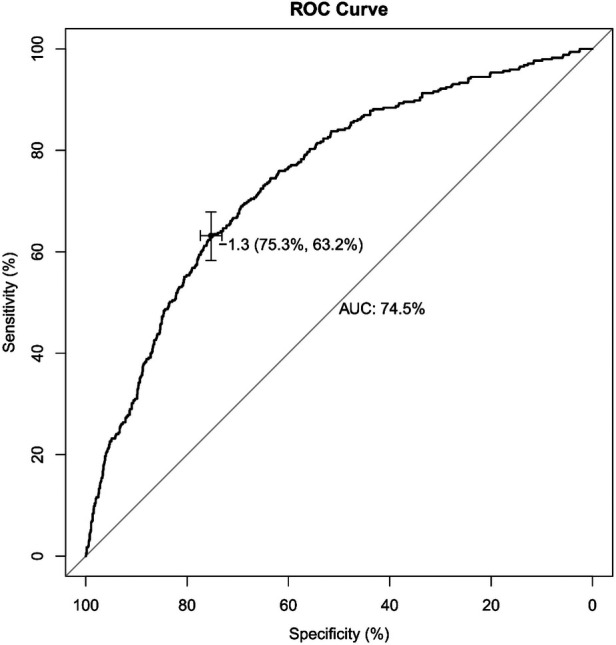
Receiver operating characteristic (ROC) curve of the nomogram model for predicting the probability of switching IS drugs. The *y*-axis represents the true positive rate of the risk prediction, and the *x*-axis represents the false positive rate of the risk prediction.

**Figure 3:**
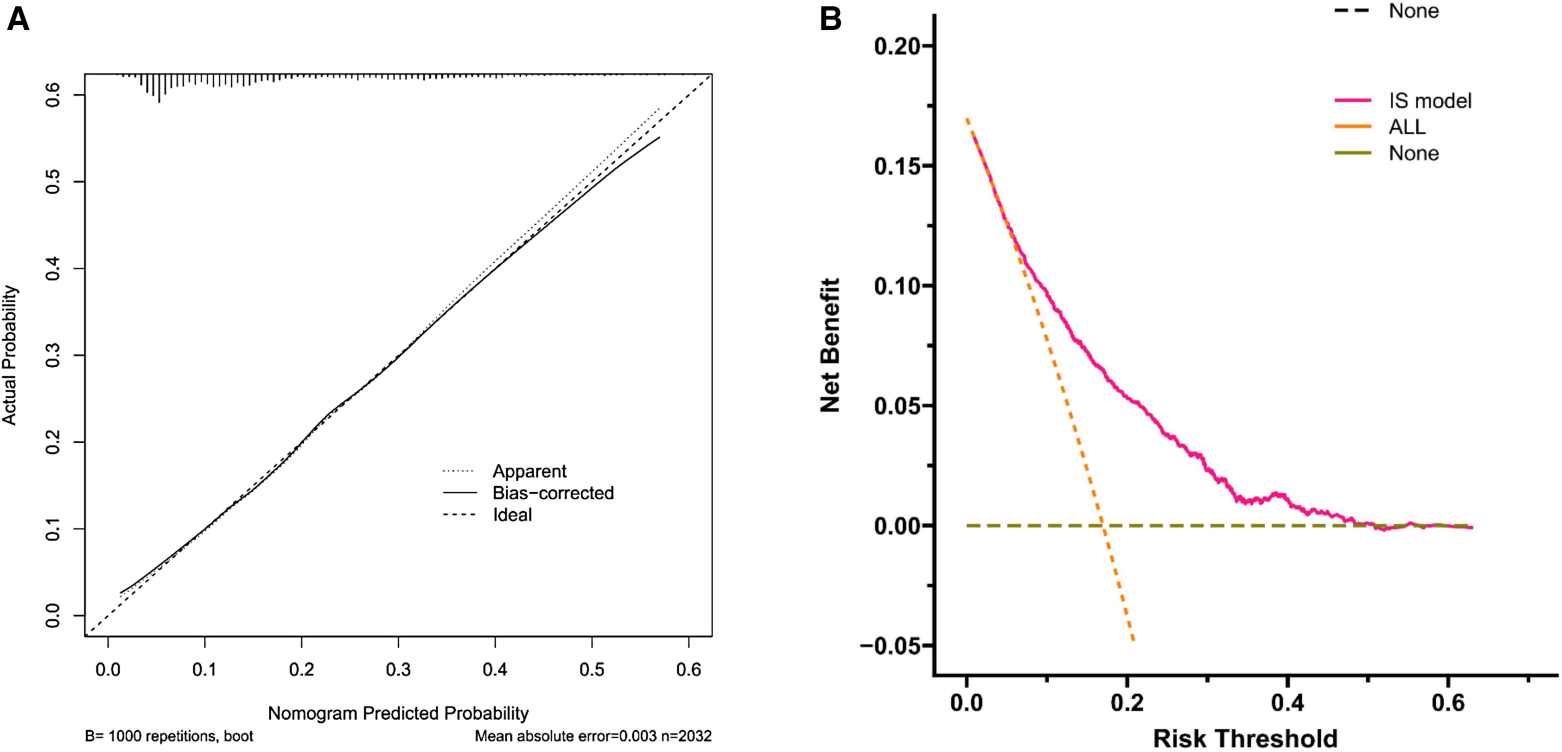
Calibration curves (**A**) comparing predicted and actual probability of switching IS drugs. The nomogram-predicted probability is shown on the *x*-axis, and the observed proportion of recipients with switching IS drugs is shown on the *y*-axis. The 45° line represents the ideal nomogram. Decision curve analysis (DCA) curves (**B**) of the nomogram. The *y*-axis shows the net benefit, while the *x*-axis shows the threshold probability. The pink line represents the nomogram, while the red and orange lines represent patients without and with switching IS drugs, respectively. The net benefit is the gap between the continuous and dotted line.

The receiver operating characteristic (ROC) curve was conducted to evaluate the prediction efficacy of the nomogram (Figure 2). The area under the ROC curve (AUC) of this nomogram was calculated at 74.5%, and the cut-off value for risk probability in this model was 1.3, with a specificity and sensitivity of 75.3% and 63.2%, respectively. The calibration curve of this nomogram for the risk between the actual and predicted probability was consistent (Figure 3A). A Decision Curve Analysis (DCA) evaluation (Figure 3B) underscored the clinical utility of the nomogram, suggesting that recipients with a threshold risk ranging from approximately 20%–50% would benefit from its utilization.

## Discussion

In this study, we developed a nomogram to predict the risk of switching IS drugs for pediatric liver transplant recipients. We used the ROC curve, calibration curve, and DCA to evaluate the model's predictive performance, which demonstrated good prediction ability with AUC values above 0.7. In interpreting the results of our study, we underscore the crucial clinical implications of our nomogram in aiding clinicians' decision-making processes. The ability to predict the risk of poor treatment outcomes for recipients undergoing a TAC-based IS regimen can empower physicians to consider alternative therapies, such as CsA, more efficiently when a poor prognosis is anticipated. This can be especially valuable in mitigating the potential side effects associated with IS drugs and optimizing patient care.

Recipients who express CYP3A4 genotype of AA may have a more difficult time achieving the target blood concentration of TAC compared to those who do not, potentially increasing the risk of TAC toxicity due to overexposure (14, 15). In our model, both recipient and donor CYP3A4 genotypes are predictors. However, it appears that recipient CYP3A4 genotype does not correlate with TAC oral clearance, as TAC primarily undergoes metabolism in the donor liver and intestine (16), and the relationship between recipient CYP3A4 genotype and TAC dosing is still unclear in pediatric liver transplantation (17). While some studies have shown that recipient CYP3A4 genotype does not significantly contribute to TAC metabolism (18), others have found it to be more prominent than donor CYP3A4 genotype (19). This discrepancy may be attributed to the association between CYP3A4 genotpy and length of time after pediatric LT and recipient age (20, 21).

The patients who were initially treated with TAC but later switched to CsA had a higher rate of acute rejection, urinary complications, and mental and neurological complications. This suggests that severe complications might have been the reason for switching TAC to CsA. However, lower mortality and incidence of portal vein complications and PTLD may indicate that some recipients benefited from switching IS drugs. CsA can be considered as an alternative to TAC-based therapy when a poor prognosis is expected and individualized therapy is needed to minimize the side effects of IS drugs.

However, we acknowledge several limitations in our study. First and foremost, our research was conducted at a single center, which may impact the generalizability of our findings. External validation from multiple transplantation centers would strengthen the reliability and applicability of our nomogram. Additionally, the presence of non-traceable missing data is an inherent limitation of retrospective studies. Future research endeavors should aim to overcome these limitations by conducting prospective studies with comprehensive data collection and validation across various clinical settings.

In conclusion, our study offers a valuable nomogram to predict the risk of unfavorable treatment outcomes for recipients undergoing a TAC-based IS regimen in pediatric liver transplantation. This tool can significantly assist clinicians in making informed decisions and improving patient care.

## Data Availability

The raw data supporting the conclusions of this article will be made available by the authors, without undue reservation.

## References

[1] Starzl TE Fung JJ. Themes of liver transplantation. Hepatology. (2010) 51:1869–84. 10.1002/hep.23595 20235333 PMC4507423

[2] Watt KD Pedersen RA Kremers WK Heimbach JK Charlton MR. Evolution of causes and risk factors for mortality post-liver transplant: results of the NIDDK long-term follow-up study. Am J Transplant. (2010) 10:1420–7. 10.1111/j.1600-6143.2010.03126.x 20486907 PMC2891375

[3] Wiesner RH Fung JJ. Present state of immunosuppressive therapy in liver transplant recipients. Liver Transpl. (2011) 17(Suppl 3):S1–9. 10.1002/lt.22410 21850697

[4] Kwong AJ Kim WR Lake JR Smith JM Schladt DP Skeans MA OPTN/SRTR 2019 annual data report: liver. Am J Transplant. (2021) 21(Suppl 2):208–315. 10.1111/ajt.16494 33595192

[5] Rana A Ackah RL Webb GJ Halazun KJ Vierling JM Liu H No gains in long-term survival after liver transplantation over the past three decades. Ann Surg. (2019) 269:20–7. 10.1097/SLA.0000000000002650 29303806

[6] Aberg F Gissler M Karlsen TH Ericzon BG Foss A Rasmussen A Differences in long-term survival among liver transplant recipients and the general population: a population-based nordic study. Hepatology. (2015) 61:668–77. 10.1002/hep.27538 25266201

[7] Mukherjee S Mukherjee U. A comprehensive review of immunosuppression used for liver transplantation. J Transplant. (2009) 2009:1–20. 10.1155/2009/701464 PMC2809333 20130772

[8] Tasdogan BE Ma M Simsek C Saberi B Gurakar A. Update on immunosuppression in liver transplantation. Euroasian J Hepatogastroenterol. (2019) 9:96–101. 10.5005/jp-journals-10018-1301 32117698 PMC7047305

[9] McAlister VC Haddad E Renouf E Malthaner RA Kjaer MS Gluud LL. Cyclosporin versus tacrolimus as primary immunosuppressant after liver transplantation: a meta-analysis. Am J Transplant. (2006) 6:1578–85. 10.1111/j.1600-6143.2006.01360.x 16827858

[10] Pflugrad H Schrader AK Tryc AB Ding X Lanfermann H Jackel E Longterm calcineurin inhibitor therapy and brain function in patients after liver transplantation. Liver Transpl. (2018) 24:56–66. 10.1002/lt.24984 29156491

[11] Rodriguez-Peralvarez M Guerrero-Misas M Thorburn D Davidson BR Tsochatzis E Gurusamy KS. Maintenance immunosuppression for adults undergoing liver transplantation: a network meta-analysis. Cochrane Database Syst Rev. (2017) 3:D11639. 10.1002/14651858.CD011639.pub2 PMC6464256 28362060

[12] Beresford T. Neuropsychiatric complications of liver and other solid organ transplantation. Liver Transplant. (2001) 7:S36–45. 10.1053/jlts.2001.29095 11689775

[13] Pruitt A. Neurological complications after solid organ transplantation. J Neurol Sci. (2015) 357:e483. 10.1016/j.jns.2015.09.241

[14] Birdwell KA Decker B Barbarino JM Peterson JF Stein CM Sadee W Clinical pharmacogenetics implementation consortium (CPIC) guidelines for CYP3A5 genotype and tacrolimus dosing. Clin Pharmacol Ther. (2015) 98:19–24. 10.1002/cpt.113 25801146 PMC4481158

[15] Fukudo M Yano I Masuda S Goto M Uesugi M Katsura T Population pharmacokinetic and pharmacogenomic analysis of tacrolimus in pediatric living-donor liver transplant recipients. Clin Pharmacol Ther. (2006) 80:331–45. 10.1016/j.clpt.2006.06.008 17015051

[16] Staatz CE Tett SE. Clinical pharmacokinetics and pharmacodynamics of tacrolimus in solid organ transplantation. Cham: Adis International (2004). 623–53. 10.2165/00003088-200443100-00001 15244495

[17] de Wildt SN van Schaik RHN Soldin OP Soldin SJ Brojeni PY van der Heiden IP The interactions of age, genetics, and disease severity on tacrolimus dosing requirements after pediatric kidney and liver transplantation. Eur J Clin Pharmacol. (2011) 67:1231–41. 10.1007/s00228-011-1083-7 21698374 PMC3214266

[18] Calvo PL Serpe L Brunati A Nonnato A Bongioanni D Olio DD DonorCYP3A5 genotype influences tacrolimus disposition on the first day after paediatric liver transplantation. Br J Clin Pharmacol. (2017) 83:1252–62. 10.1111/bcp.13219 28044353 PMC5427244

[19] Buendía JA Halac E Bosaleh A Garcia de Davila MT Imvertasa O Bramuglia G. Frequency of CYP3A5 genetic polymorphisms and tacrolimus pharmacokinetics in pediatric liver transplantation. Pharmaceutics. (2020) 12:898. 10.3390/pharmaceutics12090898 32971783 PMC7557928

[20] Thörn M Lundgren S Herlenius G Ericzon BG Lööf L Rane A. Gene expression of cytochromes P 450 in liver transplants over time. Eur J Clin Pharmacol. (2004) 60(6):413–20. 10.1007/s00228-004-0786-4 15197524

[21] Ince I Knibbe CAJ Danhof M de Wildt SN. Developmental changes in the expression and function of cytochrome P450 3A isoforms: evidence from in vitro and in vivo investigations. Clin Pharmacokinet. (2013) 52:333–45. 10.1007/s40262-013-0041-1 23463352

